# Increased Intratumoral Neutrophil in Colorectal Carcinomas Correlates Closely with Malignant Phenotype and Predicts Patients' Adverse Prognosis

**DOI:** 10.1371/journal.pone.0030806

**Published:** 2012-01-25

**Authors:** Hui-Lan Rao, Jie-Wei Chen, Mei Li, Yong-Bo Xiao, Jia Fu, Yi-Xin Zeng, Mu-Yan Cai, Dan Xie

**Affiliations:** 1 State Key Laboratory of Oncology in South China, Sun Yat-Sen University Cancer Center, Guangzhou, China; 2 Department of Pathology, Sun Yat-Sen University Cancer Center, Guangzhou, China; University of Aberdeen, United Kingdom

## Abstract

**Background:**

Substantial evidence suggests that the presence of inflammatory cells plays a critical role in the development and/or progression of human tumors. Neutrophils are the common inflammatory cells in tumors; however, the infiltration of intratumoral neutrophils in colorectal carcinoma (CRC) and its effect on CRC patients' prognosis are poorly understood.

**Methodology/Principal Findings:**

In this study, the methods of tissue microarray and immunohistochemistry (IHC) were used to investigate the prognostic significance of intratumoral CD66b+ neutrophil in CRC. According to receiver operating characteristic curve analysis, the cutoff score for high intratumoral CD66b+ neutrophil in CRC was defined when the mean counts were more than 60 per TMA spot. In our study, high intratumoral CD66b+ neutrophil was observed in 104/229 (45.4%) of CRCs and in 29/229 (12.7%) of adjacent mucosal tissues. Further correlation analysis showed that high intratumoral neutrophil was positively correlated with pT status, pM status and clinical stage (*P*<0.05). In univariate survival analysis, a significant association between high intratumoral neutrophil and shortened patients' survival was found (*P*<0.0001). In different subsets of CRC patients, intratumoral neutrophil was also a prognostic indicator in patients with stage II, stage III, grade 2, grade 3, pT1, pT2, pN0 and pN1 (*P*<0.05). Importantly, high intratumoral neutrophil was evaluated as an independent prognostic factor in multivariate analysis (*P*<0.05).

**Conclusions/Significance:**

Our results provide evidence that increased intratumoral neutrophil in CRC may be important in the acquisition of a malignant phenotype, indicating that the presence of intratumoral neutrophil is an independent factor for poor prognosis of patients with CRC.

## Introduction

Colorectal carcinoma (CRC) is a leading cause of cancer-related mortality and morbidity in the Western world, with 5-year survival rates ranging from 90% to 10% with cancer progression [1]. In China, CRC is the fifth leading cause of cancer related death and the incidence continues to increase [2]. Even among patients with CRC who undergo potentially curative resection alone, 40% to 50% of them ultimately relapse and die of metastatic disease [3]. Although tumor-nodes-metastasis (TNM) classification of CRC is a useful tool for staging CRC patients and selecting them for specific treatment, it is not sufficient, since many patients with the same TNM stage may have various outcomes, suggesting that the conventional staging procedures may be unable to precisely predict cancer prognosis [4]. Thus, a substantial amount of research on CRC has focused on the discovery of specific molecular markers that are responsible for the progression of this malignancy. To the present, however, the search and identification of promising molecular and/or genetic alterations in CRC cells that have clinical/prognostic significance remains substantially limited.

The tumor microenvironment is very important with regard to the preservation and promotion of tumor development and/or progression. Inflammation has been identified as the seventh hallmark of cancer [5]. An inflammatory milieu consisting of immune/inflammatory cell and their secretory products can promote tumor progression. The type, density and location of tumor-infiltrating immune cells in the local microenvironment have been related to the clinical outcome of several types of human cancers [6], [7]. Human neutrophils, initially recognized as key effectors in the first-line host defense against invading pathogens, are the most abundant subpopulation of leucocytes [8], [9]. In addition to direct bactericidal activities, neutrophils can actively regulate angiogenesis and tissue remodeling by releasing multiple proteases [8], [10]. Increased levels of neutrophils have been found in various human tumors, and studies in mice indicated that, depending on microenvironment, tumor-infiltrating neutrophils are capable of being pro-tumor effect [11], [12], [13]. Immune cells within the tumor tissues have been found to be a better predictor of patient survival than the histopathological methods currently used to stage CRC [6]. In addition, an elevated neutrophil-to-lymphocyte ratio (NLR>5) of the peripheral blood, reflecting the systemic immune response, has been correlated with poor clinical outcome in patients with advanced CRC [14]. However, the role of tumor-infiltrating neutrophils in the local CRC microenvironment remains to be elucidated.

Since CD66b was reported to be specifically expressed in neutrophils [15] and it has been widely used to investigate the neutrophil infiltration in different types of human cancer, such as renal cell, hepatocellular and nonsmall cell cancers and melanoma [12], [16], [17], [18]. In the present study, therefore, the IHC staining of CD66b was utilized to investigate the clinical/prognostic significance of intratumoral neutrophils in a cohort of CRCs. Herein, we report, for the first time, that increased intratumoral neutrophils, as detected by IHC, correlate closely with CRC malignant phenotype and is an independent predictor for shortened survival time of patients with CRC.

## Materials and Methods

### Ethics statement

The study was approved by the Institute Research Medical Ethics Committee of Sun Yat-Sen University. No informed consent (written or verbal) was obtained for use of retrospective tissue samples from the patients within this study, most of whom were deceased, since this was not deemed necessary by the Ethics Committee, who waived the need for consent. All samples were anonymised.

### Patients and tissue specimens

In this study, the paraffin-embedded pathologic specimens from 229 patients with CRC were obtained from the archives of Department of Pathology, Sun Yat-Sen University Cancer Center and Guangdong Provincial People's Hospital, Guangzhou, China, between January 2000 and November 2006. All these resection samples have a uniform fixation, dissection and processing protocol. The cases selected were based on distinctive pathologic diagnosis of CRC, undergoing primary and curative resection for CRC, availability of resection tissue, follow-up data, and had not received preoperative anticancer treatment. These CRC cases included 142 (62.0%) men and 87 (38.0%) women, with mean age of 57.3 years. Average follow-up time was 55.42 months (median, 60.0 months; range, 0.5 to 98 months). These patients, whose diseases encompassed 171 (74.7%) colon cancers (including 89 left-sided colon and 82 right-sided colon cancers) and 58 (25.3%) rectal cancers, underwent initial surgical treatment in our institute. The mean lymph node yield for each resected colorectal cancer was 15. The diagnostic examinations including MRI, CT, chest X-ray, abdominal ultrasonography and bone scan were utilized to identify CRC distal metastasis. The pTNM status of all CRCs was assessed according to the criteria of the sixth edition of the TNM classification of the International Union Against Cancer (UICC, 2002). Clinicopathologic characteristics for these patients were detailed in table 1. Patients whose cause of death remained unknown or received preoperative radiation or chemotherapy were excluded from our study. In addition, for analyzing the levels of neutrophil infiltration in CRC lymph node metastasis, 30 cases of lymph node metastatic samples from our CRC cohort were selected and studied.

**Table 1:** Correlation between the clinicopathologic variables and intratumoral CD66b+ neutrophil in colorectal carcinoma.

		Intratumoral CD66b+ neutrophil		
	All cases	Low	High	*P* value*
Sex				0.492
Female	87	50 (57.5%)	37 (42.5%)	
Male	142	75 (52.8%)	67 (47.2%)	
Age at diagnosis (years)				0.990
≤57.3†	108	59 (54.6%)	49 (45.4%)	
>57.3	121	66 (54.5%)	55 (45.5%)	
Tumor location				0.084
Colon	171	99 (57.9%)	72 (42.1%)	
Rectum	58	26 (44.8%)	32 (55.2%)	
Histological grade (WHO)				0.264
G1	19	13 (68.4%)	6 (31.6%)	
G2	174	90 (51.7%)	84 (48.3%)	
G3	36	22 (61.1%)	14 (38.9%)	
pT status				0.039
1	16	13 (81.3%)	3 (18.7%)	
2	76	44 (57.9%)	32 (42.1%)	
3	129	66 (51.2%)	63 (48.8%)	
4	8	2 (25.0%)	8 (75.0%)	
pN status				0.258
0	169	96 (56.8%)	73 (43.2%)	
1	60	29 (48.3%)	31 (51.7%)	
pM status				0.001
pMX	206	120 (58.3%)	86 (41.7%)	
pM1	23	5 (21.7%)	18 (78.3%)	
Clinical stage				0.002
I	74	49 (66.2%)	25 (33.8%)	
II	76	44 (57.9%)	32 (42.1%)	
III	56	27 (48.2%)	29 (51.8%)	
IV	23	5 (21.7%)	18 (78.3%)	

*Chi-square test;

† Mean age;

WHO indicated World Health Organization.

### Tissue microarray (TMA) and immunohistochemistry (IHC)

TMAs were constructed in accordance with a previously-described method [19]. Triplicate 0.6 mm diameter cylinders from the center of the tumor away from areas of ulceration/necrosis were punched from representative areas of an individual donor tissue block, and re-embedded into a recipient paraffin block in a defined position, using a tissue arraying instrument (Beecher Instruments, Silver Spring, MD).

The TMA blocks were cut into 5-µm sections and processed for IHC in accordance with a previously-described protocol [20]. TMA slides were incubated with anti-CD66b ((BD Transduction Laboratories, Franklin Lakes, NJ, 1∶1000 dilution) and anti-CD3 (Novocastra, Newcastle upon Tyne, UK, 1∶100 dilution), respectively, and stored overnight at 4°C. Immunostaining was performed using the Envision System with diaminobenzidine (Dako, Glostrup, Denmark). A negative control was obtained by replacing the primary antibody with a normal murine or rabbit IgG. In the case of non-informative TMA samples (i.e., samples with <500 tumor cells per case and lost samples), IHC staining was performed by using whole tissue slides.

### IHC evaluation

Microarray was evaluated at 200× magnification light microscopy by two independent pathologists (H-L. Rao and M-Y. Cai) who were blinded to the clinicopathological data of these CRC patients. For CD66b and CD3 IHC staining, positive cells in each 0.6-mm-diameter cylinder were counted and expressed as mean value of the triplicates.

### Selection of cutoff score

**Figure 1:**
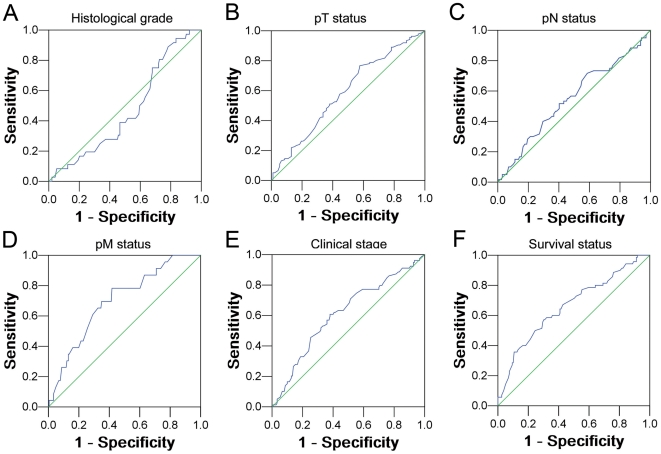
ROC curve analysis was employed to determine the cutoff value for high intratumoral CD66b+ neutrophil in colorectal carcinoma. The sensitivity and specificity for each outcome were plotted: tumor grade (*P* = 0.575, A), T stage (*P* = 0.029, B), N stage (*P* = 0.263, C), M stage (*P* = 0.002, D), clinical stage (*P* = 0.005, E), and survival status (*P*<0.001, F).

Receiver operating characteristic (ROC) curve analysis was performed to determine cutoff score for tumor with increased intratumoral neutrophil by using the 0,1-criterion [21]. At the intratumoral neutrophil counts, the sensitivity and specificity for each outcome under study was plotted, thus generating various ROC curves (Figure 1). The count was selected as the cutoff value, which was closest to the point with both maximum sensitivity and specificity. Tumors designated as decreased intratumoral neutrophils were those with the scores below or equal to the cutoff value, while tumors of increased intratumoral neutrophils were those with scores above the value. In order to use ROC curve analysis, the clinicopathological features were dichotomized: histological grade (G1–G2 or G3), pT status (T1–T2 or T3–T4), pN status (N0 or N1), pM status (pMX or pM1), clinical stage (I+II or III+IV) and survival (death due to CRC or censored [lost to follow-up, alive or death from other causes]).

### Statistical analysis

Statistical analysis was performed by using the SPSS statistical software package (standard version 13.0; SPSS, Chicago, IL). ROC curve analysis was applied to determine the cutoff score for increased intratumoral neutrophils and T lymphocytes. The correlation between increased intratumoral neutrophils and clinicopathological features of CRC patients was analyzed by χ^2^-test. For univariate survival analysis, survival curves were obtained with the Kaplan-Meier method. The Cox proportional hazards regression model was used to identify the independent prognostic factors. Differences were considered significant if the *P*-value from a two-tailed test was less than 0.05.

## Results

### The pattern of CD66b+ neutrophil in colorectal tissues

**Figure 2:**
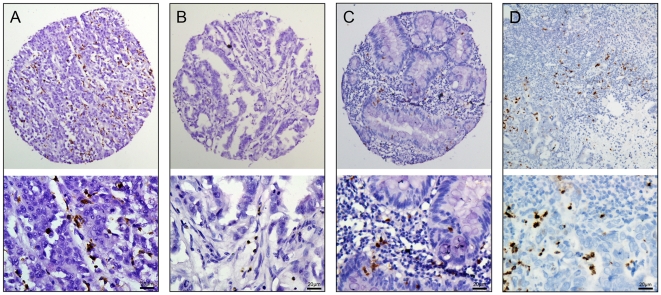
The presence of CD66b+ neutrophils in colorectal and lymph node metastatic CRC tissues. (A) High intratumoral neutrophil was observed in a CRC (case 52), in which more than 60 intratumoral cells revealed positive immunostaining of CD66b (*upper panel*, ×100). (B) A CRC case (case 37) demonstrated low intratumoral neutrophil, in which less than 60 intratumoral cells showed immunoreactivity of CD66b (*upper panel*, ×100). (C) The corresponding adjacent mucosal tissue of case 52 showed low neutrophil, in which less than 60 cells revealed immunostaining of CD66b (*upper panel*, ×100). (D) High intratumoral neutrophil was observed in lymph node metastatic tissue, in which more than 60 intratumoral cells revealed positive immunostaining of CD66b (*upper panel*, ×100). The *lower panels* indicated the higher magnification (×400) from the area of the box in A, B and C, respectively.

To investigate the pattern of neutrophil in carcinomatous and non-neoplastic human colorectal tissues, we conducted IHC staining of CD66b on a CRC-TMA containing 229 pairs of primary CRC specimens and corresponding normal colorectal mucosa, and on 30 samples (whole tissue slides) of CRC lymph node metastasis. We examined that CD66b+ neutrophils were localized primarily in the stroma of CRC and colorectal mucosal tissues. IHC staining of CD66b in representative samples of CRC, adjacent normal colorectal mucosa and lymph node metastatic tissues were shown in Figure 2. The counts of intratumoral CD66b+ neutrophil in CRC tissues ranged from 0 to 135.0 per TMA spot, with median count of 45.0.

### Selection of cutoff score for high intratumoral CD66b+ neutrophil in CRC

The ROC curves for each clinicopathologic feature (Figure 1) show the value on the curve closest to the point (i.e., 0.0, 1.0), which maximizes both sensitivity and specificity for the outcome [21]. Tumors with counts above the obtained cutoff value were considered as high intratumoral neutrophil leading to the greatest number of tumors correctly classified as having or not having the clinical outcome. The corresponding area under the curve (AUC) were collected and shown in Table 2. In our current study, ROC curve analysis for pM status had the shortest distance from the curve to the point (i.e., 0.0, 1.0), and we selected the cutoff value determined by pM status. Thus, the cutoff score for high intratumoral CD66b neutrophil in CRC was defined when the mean counts were more than 60 per TMA spot.

**Table 2:** Area under the receiver (AUC) operating characteristic curve for each clinicopathological feature.

Feature	AUC (95% CI)	*P* value
Grade	0.471 (0.377 to 0.564)	0.575
T stage	0.585 (0.510 to 0.661 )	0.029
N stage	0.549 (0.462 to 0.635)	0.263
M stage	0.699 (0.594 to 0.805)	0.002
Stage	0.612 (0.535 to 0.689)	0.005
Survival	0.662 (0.584 to 0.740)	<0.001

CI indicates confidence interval.

### The association of the increased intratumoral neutrophil with CRC patients' clinicopathologic features

According to ROC curve, high intratumoral CD66b+ neutrophil was examined in 29/229 (12.7%) of normal colorectal mucosal tissues, in 104/229 (45.4%) of primary CRC tissues and in 16/30 (53.3%) of lymph node metastatic CRC tissues (*P*<0.001, Chi-square test). There is no significant difference of CD66b+ neutrophil infiltration in primary CRC and lymph node metastatic CRC tissues (*P* = 0.413). The rates of high intratumoral neutrophil in CR with respect to several standard clinicopathologic features were detailed in Table 1. The results showed that increased intratumoral neutrophil was positively correlated with tumor pT status, pM status and advanced clinical stage (*P*<0.05, Table 1). There was no significant association between intratumoral CD66b+ neutrophil and other clinicopathologic features, such as patient gender, age, histological grade and pN status (*P*>0.05, Table 1).

### Association between clinicopathologic characteristics, intratumoral CD66b+ neutrophil, and CRC patient survival: univariate survival analysis

**Figure 3:**
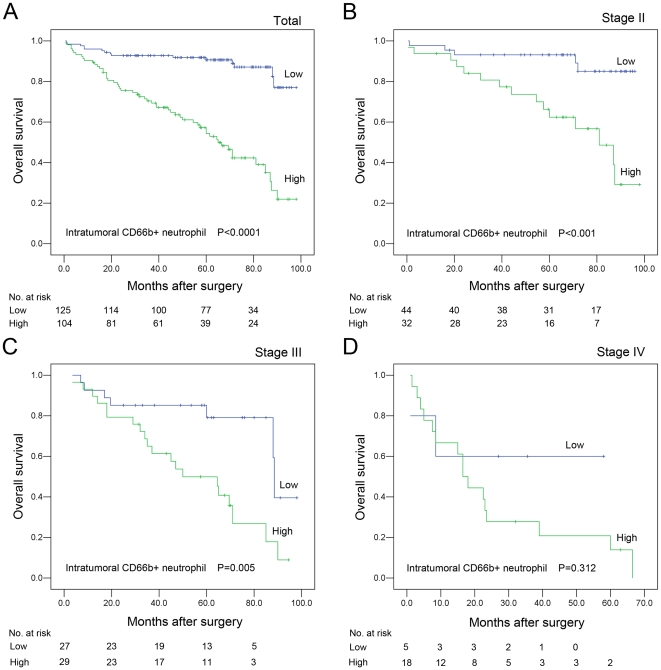
Survival curves for 229 CRC patients according to infiltration status of intratumoral neutrophils (log-rank test). (A) *Total*, probability of survival of all patients with CRC: low intratumoral neutrophil, n = 125; high intratumoral neutrophil, n = 104. (B) *Stage II*, probability of survival of stage II patients with CRC: low intratumoral neutrophil, n = 44; high intratumoral neutrophil, n = 32. (C) *Stage III*, probability of survival of stage III patients with CRC: low intratumoral neutrophil, n = 27; high intratumoral neutrophil, n = 29. (D) *Stage IV*, probability of survival of stage IV patients with CRC: low intratumoral neutrophil, n = 5; high intratumoral neutrophil, n = 18.

**Figure 4:**
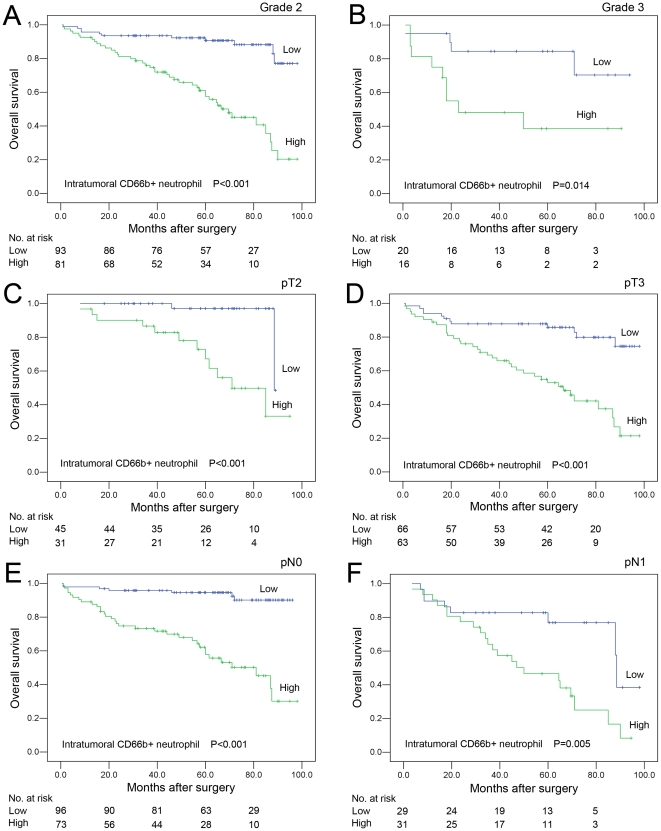
Survival curves according to infiltration status of intratumoral neutrophils in subsets of CRC patients with different histological grade and pT/pN status (log-rank test). (A) *Grade 2*, probability of survival of grade 2 patients with CRC: low intratumoral neutrophil, n = 93; high intratumoral neutrophil, n = 81. (B) *Grade 3*, probability of survival of grade 3 patients with CRC: low intratumoral neutrophil, n = 20; high intratumoral neutrophil, n = 16. (C) *pT2*, probability of survival of pT2 patients with CRC: low intratumoral neutrophil, n = 45; high intratumoral neutrophil, n = 31. (D) *pT3*, probability of survival of pT2 patients with CRC: low intratumoral neutrophil, n = 66; high intratumoral neutrophil, n = 63. (E) *pN0*, probability of survival of pN0 patients with CRC: low intratumoral neutrophil, n = 96; high intratumoral neutrophil, n = 73. (F) *pN1*, probability of survival of pN1 patients with CRC: low intratumoral neutrophil, n = 29; high intratumoral neutrophil, n = 31.

To confirm the representativeness of the CRC cohort in our study, we first tested well-established prognostic factors of patient survival. Kaplan–Meier analysis evaluated a significant impact of well-known clinicopathologic prognostic parameters, such as pT status (*P* = 0.012), pN status (*P*<0.0001), pM status (*P*<0.0001), and clinical stage (*P*<0.0001) on patients' survival (Table 3). Assessment of survival in our CRC cohort demonstrated that the increased intratumoral CD66b+ neutrophil was correlated with adverse disease-specific survival (*P*<0.0001, Figure 3A and Table 3). Moreover, survival analysis was performed with regard to intratumoral CD66b+ neutrophil infiltration in subsets of CRC patients with different clinical stage (Figure 3B–3D), histological grade (Figure 4A and 4B) and pT/pN status (Figure 4C–4F). Our results demonstrated as well that high intratumoral neutrophil was a prognostic factor in CRC patients with stage II (*P*<0.001, Figure 3B), stage III (*P* = 0.005, Figure 3C), grade 2 (*P*<0.001, Figure 4A), grade 3 (*P* = 0.014, Figure 4B), pT1 (*P*<0.001, Figure 4C), pT2 (*P*<0.001, Figure 4D), pN0 (*P*<0.001, Figure 4E) and pN1 (*P* = 0.005, Figure 4F). But it could not differentiate the outcome of stage I (not reached), stage IV patients (*P*>0.05, Figure 3D), grade 1 (not reached), pT1 (not reached), or pT4 (*P*>0.05, data not shown).

**Table 3:** Univariate and multivariate analysis of different prognostic factors in 229 patients with colorectal carcinoma.

Variable	Univariate analysis*				Multivariate analysis†	
	All cases	Mean survival (months)	Chi-square value	*P* value	HR (95% CI)	*P* value
Sex			0.176	0.675		
Female	87	76.7				
Male	142	74.6				
Age at surgery (years)			0.005	0.943		
≤57.3‡	108	74.5				
>45	121	75.5				
Tumor location			0.161	0.688		
Colon	171	76.1				
Rectum	58	72.4				
Histological grade (WHO)			2.291	0.130		
G1–2	193	77.4				
G3	36	64.8				
pT status			12.466	0.000		0.026
T1–T2	92	84.9			1.0	
T3–T4	137	68.7			1.951 (1.084–3.511)	
pN status			14.216	0.000		0.387
N0	169	80.3			1.0	
N1–N2	60	63.4			1.578 (0.562–4.427)	
pM status			75.992	0.000		0.283
pMX	206	80.5			1.0	
pM1	51	28.2			2.594 (0.455–14.784)	
Clinical stage			48.891	0.000		0.336
I–II	150	86.5			1.0	
III–IV	79	55.4			1.558 (0.632–3.843)	
Intratumoral CD66b+ neutrophil			48.675	0.000		0.010
Low	125	88.8			1.0	
High	104	60.0			2.040 (1.186–3.507)	

*Log-rank test;

† Cox regression model;

‡ Mean age;

HR indicates hazards ratio; CI indicates confidence interval; WHO indicates World Health Organization.

### Independent prognostic factors of CRCs: multivariate survival analysis

Since variables observed to have prognostic influence by univariate analysis may covariate, the count of intratumoral neutrophil as well as other clinicopathologic features (pT status, pN status, pM status and clinical stage) that were significant in univariate analysis were analyzed in multivariate analysis (Table 3). We found that the increased intratumoral CD66b+ neutrophil was evaluated as an independent risk factor for adverse overall patient survival (hazards ratio: 2.040; 95% confidence interval: 1.186–3.843; *P* = 0.010). Of the other variables, pT status was also found to be an independent prognostic predictor for overall survival (Table 3).

### Correlation between the neutrophil and T cell infiltration in CRCs

Similarly, by utilizing the ROC curve analysis, the cutoff score for high intratumoral CD3+ T lymphocyte in CRC was defined when the mean counts were more than 76 per TMA spot. High intratumoral CD3+ T cell was detected in 112/229 (48.9%) of CRCs. Further correlation analysis showed that there was no statistically significant correlation between expression of neutrophil and T lymphocyte infiltration in our CRC cohort (*P* = 0.144, Fishers exact test).

## Discussion

It has been long recognized that many human cancer types are accompanied by inflammatory cell infiltration of varying intensity. Most attention has been paid to a possible role of inflammatory cells, due to their obvious immunological correlation. Recent studies have elucidated the role of distinct immune cells, cytokines, and other immune mediators in virtually all steps of colorectal tumorigenesis, including initiation, promotion, progression and metastasis. In addition, the type, density and location of T lymphocytes, CD8 T cell effectors and their associated cytotoxic molecule, and memory T cells in CRCs had a prognostic value that was superior to and independent of the TNM classification [6]. However, up to the present, the role of intratumoral neutrophils in CRCs has not been well understood.

In the presents study, we utilized the methods of TMA and IHC to investigate the clinicopathologic significance of the intratumoral CD66b+ neutrophils in CRC tissues. Our current study established that neutrophils could be detected in most of intratumoral stroma of CRC and lymph node metastatic tissues by IHC, and that increased intratumoral neutrophil was positively correlated with CRC pT status, pM status and advanced clinical stage. Moreover, univariate and multivariate analyses evaluated that increased intratumoral neutrophil was a prognostic factor independent of certain well-established clinical features, including tumor pT status, pN status, pM status and clinical stage. Thus,in this study, our results provided evidence that increased intratumoral neutrophil in CRC may facilitate an increased malignant and/or worse prognostic phenotype of the tumor.

As a canonical leucocyte subpopulation, human neutrophils have been recognized as a first-line defender against infectious microorganisms. However, neutrophils are a source of N-nitrosamines, especially in the presence of colonic amine producing bacteria, and these are known to be carcinogenesis [22], [23]. Campreqher et al [24] reported that activated neutrophils induced an hMSH2-dependent G2/M checkpoint arrest and replication errors in colon epithelial cells. Previous study showed that infiltration of neutrophils was increased in colorectal adenomas compared to adjacent normal mucosa and correlated with adenoma size, suggesting that the presence of neutrophils is involved in the early stage of colorectal tumorigenesis [25]. Recently, expression of neutrophil gelatinase-associated lipocalin (NGAL) has been shown to be elevated in both colorectal adenoma-carcinoma sequence and cancer progression and enhances tumorigenesis in mouse models [26]. Also, overexpression of NGAL in CRCs is an important regulatory molecule that integrates extracellular environment cues, iron metabolism, and intracellular small GTPase signaling in cancer migration and invasion [27]. These findings are in line with our observations, suggesting intratumoral neutrophils as an adverse prognostic factor for CRC.

In our study, moreover, increased intratumoral CD66b+ neutrophil was also frequently found in CRC lymph node metastatic tissues and it was positively correlated to the metastatic phenotype of CRC. Neutrophil elastase, is a main constituent of phagocytic response, degrading proteins within the local environment such as elastin, collagen and other constituents of extracellular matrix [28]. It was demonstrated that neutrophil and macrophage derived MMP-9 enhanced progression from dysplasia to overt malignancy in a mouse model of skin squamous carcinoma, with reduced MMP-9 delaying angiogenesis within dysplastic areas and reducing the incidence of invasive disease [29]. Moreover, it has been suggested that the accumulated neutrophils in the peritumoral stroma of HCC are the major source of MMP-9, which in turn trigger the angiogenic switch at the adjacent invading edge [30]. These data, taken together, might provide a possible explanation that in our present study, why the increased intratumoral neutrophil was positively correlated with distant metastasis in CRCs.

The impact of neutrophil infiltrates on the prognosis in CRC patients remained controversial. Nielsen et al found that the high peritumoral neutrophil cell counts had a good prognostic impact on CRC patients by assessing H&E staining slides [31]. In our study, the increased intratumoral CD66b+ neutrophil was associated closely with CRC patients' shorter survival time and evaluated as an independent risk factor for poor overall survival. Other groups reported that the presence of intratumoral neutrophils was an adverse prognostic factor for HCC after resection [16]. In addition, accumulation of neutrophils in peritumoral stroma was found to foster disease progression and predict reduced survival in HCC patients [30]. Furthermore, Jensen and colleagues [12] observed that neutrophil infiltration in renal cell carcinoma (RCC) specimens was correlated with poor prognosis in localized RCC. Taken together, CRC cells often recruit a wide variety of inflammatory cells to their microenvironment, resulting in tumor progression; while in peritumoral stroma, the peritumoral neutrophils might provide a protective role to inhibit CRC growth.

Thus, the examination of intratumoral neutrophil in CRCs, could be used as an additional effective tool in identifying those CRC patients at increased risk of tumor metastasis and/or progression. This might also aid the clinician to select a suitable therapy for the individual patient, e.g. favoring a more aggressive regimen in tumors with an increased intratumoral neutrophil.
